# Electrostatic potential-derived charge: a universal OER performance descriptor for MOFs†

**DOI:** 10.1039/d2sc04898a

**Published:** 2022-10-18

**Authors:** Xiangdong Xue, Hongyi Gao, Jiangtao Liu, Ming Yang, Shihao Feng, Zhimeng Liu, Jing Lin, Jitti Kasemchainan, Linmeng Wang, Qilu Jia, Ge Wang

**Affiliations:** Beijing Advanced Innovation Center for Materials Genome Engineering, Beijing Key Laboratory of Function Materials for Molecule & Structure Construction, School of Materials Science and Engineering, University of Science and Technology Beijing Beijing 100083 PR China hygao@ustb.edu.cn gewang@ustb.edu.cn; Shunde Graduate School, University of Science and Technology Beijing Shunde 528399 PR China; State Key Laboratory of Advanced Chemical Power Sources, Guizhou Meiling Power Sources Co., Ltd. Zunyi Guizhou 563003 PR China; Department of Applied Physics, The Hong Kong Polytechnic University Hung Hom Hong Kong SAR China; Department of Chemical Technology, Chulalongkorn University Bangkok 10330 Thailand

## Abstract

Metal–organic frameworks (MOFs) provide opportunities for the design of high-efficiency catalysts attributed to their high compositional and structural tunability. Meanwhile, the huge number of MOFs poses a great challenge to experimental-intensive development of high-performance functional applications. By taking the computationally feasible and structurally representative trigonal prismatic secondary building units (SBUs) of MOFs as the entry point, we introduce a descriptor-based approach for designing high-performance MOFs for the oxygen evolution reaction (OER). The electrostatic potential-derived charge (ESPC) is identified as a robust and universal OER performance descriptor of MOFs, showing a distinct linear relationship with the onset potentials of OER elemental steps. Importantly, we establish an ESPC-based physical pattern of active site–intermediate binding strength, which interprets the rationality of ESPC as an OER performance descriptor. We further reveal that the SBUs with Ni/Cu as active site atoms while Mn/Fe/Co/Ni as spectator atoms have excellent OER activity through the variation pattern of ESPC along with metal composition. The universal correlation between ESPC and OER activity provides a rational rule for designing high-performance MOF-based OER electrocatalysts and can be easily extended to design functional MOFs for a rich variety of catalytic applications.

## Abstract 2

The electrostatic potential-derived charge (ESPC) is identified as a robust and universal OER performance descriptor of MOFs. By using ESPC as a bridge, the composition design principle of MOFs can be easily derived.

## Graphical abstract

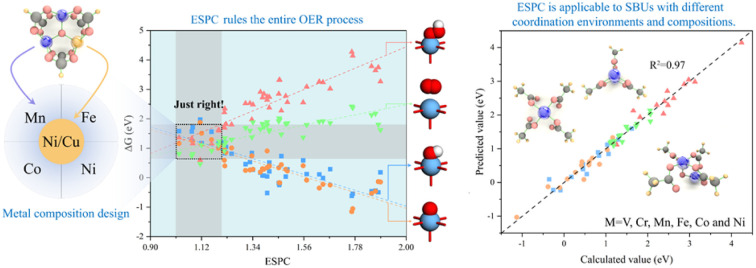

## 1. Introduction

Metal–organic frameworks (MOFs) are a class of materials assembled from SBUs and organic ligands according to a certain topological structure.^1,2^ Owing to their nature of large specific surface area, penetrating pore structures, abundant and atomically dispersed coordination unsaturated active sites (CUSs), flexible tunability of the composition and structure, *etc.*, these materials have been extensively studied in molecular storage/sieving,^3–7^ catalysis,^8–10^ sensors,^11–13^ batteries,^14,15^ and phase transitions.^16–18^ During the past six years, over 100 000 MOFs have been collected in the Cambridge Structural Database (CSD)^19,20^ and the number keeps on increasing dramatically. This, however, makes it challenging for conventional trial-and-error experiments to identify promising MOFs for high-performance functional applications.

The development of high-throughput computational methods has provided a solution to screen high-performance MOFs efficiently and effectively.^21–31^ Many studies have focused on the screening of gas storage/sieving,^28–30^ using computationally cheap characterization for the relevant geometry (pore size distributions, pore diameters, surface area, pore volume, *etc.*) and molecular-level Grand Canonical Monte Carlo (GCMC)/molecular dynamics (MD) simulations. These efforts have greatly contributed to the development of high-performance MOFs by screening promising materials from tens of thousands or even half a million MOF structures. However, high-throughput computational screening is also called “brute-force screening,”^32^ meaning that it can only handle low-cost molecular-level simulations. Even with high-performance supercomputers, it is still difficult to deal with expensive electronic-level calculations. There are few electronic-level screening efforts involving specific catalytic reaction such as photocatalysis^27^ or thermocatalysis.^22–26^ Due to the costly quantum mechanics-based computational approach, the electronic structure or reaction barrier can only be calculated for a few tens to hundreds of MOFs even using an optimized workflow.

Herein, in order to break the barrier of traditional “brute-force screening” and achieve rational design of MOFs with little computational cost, we develop a promising descriptor-based pathway. Specifically, the pivotal reaction driving the transformation of energy structure, like the OER, was chosen as the target reaction, and the secondary building units (SBUs) that play a decisive role in catalytic performance were selected as a rational simplified scheme for MOFs. In particular, we selected the widespread trigonal prismatic SBUs possessing a small number of atoms as a case, whose characteristics such as universal atomic 5-coordination configuration and 3-metal coupling endow them with excellent transferability and compositional tunability, making them an ideal platform for mining descriptive models. With monometallic SBUs, we identify ESPC as a highly accurate, instructive, transferable, and readily available OER performance descriptor. The descriptor was then employed to predict the OER activity of SBUs with bimetallic composition, and the accuracy of the descriptive model was improved using DFT-derived data. Furthermore, by correlating ESPC with OER performance of SBUs with different structures and compositions, a universal linear scaling relationship between ESPC and OER performance was identified, illustrating the universality of ESPC as an OER performance descriptor. For the purpose of using ESPC to guide the actual experimental synthesis, we deeply analyzed the relationship between ESPC and bond order as well as band structure, established an ESPC-based physical pattern of active site–intermediate binding strength. Moreover, the variation regularity of ESPC with metal composition was revealed, which demonstrated that the MOFs with Ni/Cu as active sites and Mn/Fe/Co/Ni as spectator atoms have excellent OER activity.

## 2. Methods

Spin-polarized density functional theory (DFT) calculations were performed by using the linear combination of atomic orbitals (LCAO) method embedded in DMol^3^. A polarized basis set of double numerical polarization (DNP)^33^ was employed to ensure the computational accuracy as well as the descriptions of the hydrogen bond. The electronic exchange–correlation effect was described by the Perdew–Burke–Ernzerhof (PBE)^34^ functional of general gradient approximation (GGA) level. The large orbital cutoff of 6 Å was chosen for good convergence, and TS^35^ method was adopted for a long-range van der Waals (vdW) correction. For description of the electron–ion interaction, the DFT-based relativistic semi-core pseudopotentials (DSPPs)^36^ were adopted. For reliable adsorption energy, the basis set superposition error (BSSE) correction^37^ was adopted, by specifying the catalysts as Counterpoise 1 and adsorbed O-contained species as Counterpoise 2. During the geometric optimizations, all atoms were allowed to relax until the models reach the ground state without any imaginary frequency. The convergence criteria for self-consistent field (SCF) iteration and the max force on each atom were 10^−8^ Ha and 0.001 Ha Å^−1^, respectively.

The method for bond order (BO) calculation used in this work was given by Mayer in 1986:^38^1
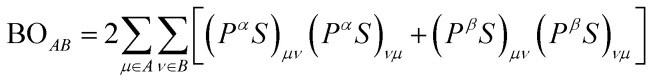
where BO_*AB*_ is the bond order between atom *A* and atom *B*, *P*^*α*^ and *P*^*β*^ are the density matrices for spin *α* and *β*, and *S* is the overlap matrix. The BOs obtained by this method are close to the corresponding classical values, and unlike Mulliken BOs, Mayer quantities are less dependent on the basis set choice and they are transferable, so they can be used to describe similar systems.

The widely studied four-electron mechanism is used to simulate the entire OER process:^39,40^* + OH^−^ → HO* + e^−^, Δ*G*_1_HO* + OH^−^ → O* + H_2_O + e^−^, Δ*G*_2_O* + OH^−^ → HOO* + e^−^, Δ*G*_3_HOO* + OH^−^ → * + H_2_O + O_2_ + e^−^, Δ*G*_4_where the * represents the bare catalyst while the HO*, O*, and HOO* denote different intermediates adsorbed on the catalyst, respectively. For each elemental step, Δ*G*_*i*_ (*i* = 1, 2, 3, 4) is the change of Gibbs energy and can be expressed as follows:^41^Δ*G* = Δ*E* + ΔZPE − *T*Δ*S*where Δ*E* is the reaction energy calculated by DFT, ΔZPE represents the change of zero-point energy under the harmonic approximation, and *T*Δ*S* represents the entropy correction. The computational hydrogen electrode (CHE) model (*G*(H^+^ + e^−^) = 1/2*G*(H_2_)) proposed by Nørskov^42–44^ was adopted to calculate the Gibbs energy of proton–electron pairs (H^+^ + e^−^). We used the *G*(O_2_) = 4.92 + 2*G*(H_2_O) − 2*G*(H_2_) to derive the free energy of triplet oxygen molecule since the energy calculated by DFT is inaccurate.^45^

Based on the above Gibbs energy results, the Gibbs energy difference of the potential-limiting step is defined as follows:Δ*G*_MAX_ = max{Δ*G*_1_, Δ*G*_2_, Δ*G*_3_, Δ*G*_4_}

## 3. Results and discussion

### 3.1 Construction of an ESPC-based OER model

**Fig. 1:**
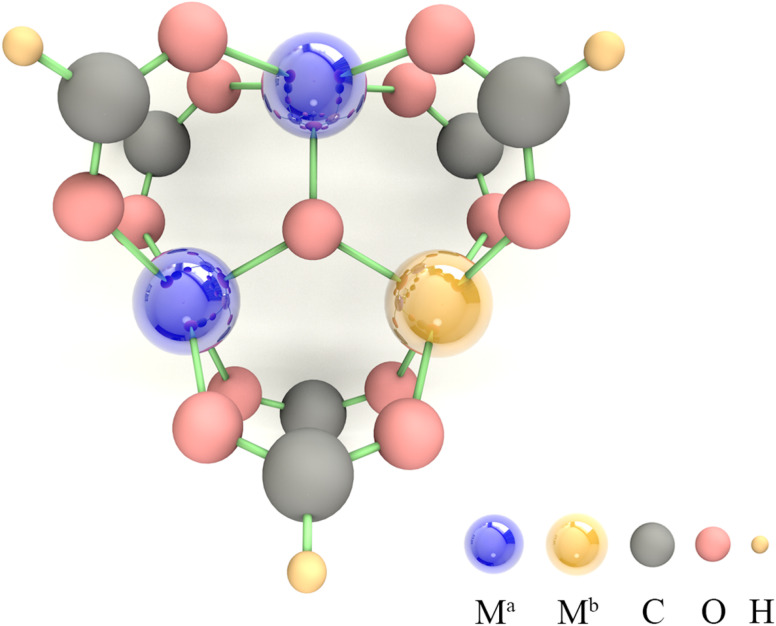
The model of the trigonal prismatic secondary building unit used in the DFT calculations, where M^a^ = V, Cr, Mn, Fe, Co, and Ni; M^b^ = V, Cr, Mn, Fe, Co, Ni, and Cu.

Due to the limitations imposed by the large number of atoms in MOFs for periodic DFT calculations, SBUs, which play a decisive role in the catalytic process, are usually selected as an alternative to MOFs for calculation.^46,47^ Among them, trigonal prismatic SBUs are a common class of SBUs (Fig. 1), whose few atoms make themselves feasible to be implemented in high-throughput calculations. In addition, the strong interaction of three metal atoms connected by a μ_3_ oxygen atom facilitates the fine-tuning of electronic structure by component orthogonalization. Moreover, the 6-coordinated metal atoms can readily desorb solvent molecules upon activation to form 5-coordinated unsaturated active sites that are widely present in MOFs, making the calculation-derived conclusions of such SBUs highly transferable. Therefore, we chose the trigonal prismatic SBUs as an entry point to study the general principles for the design of MOFs with high OER performance. In this study, the SBUs with monometallic components are labeled as M^a^ (M^a^ = V, Cr, Mn, Fe, Co, and Ni), and the SBUs with bimetallic components are labeled as M^a^2M^b^1 (M^a^ = V, Cr, Mn, Fe, Co and Ni; M^b^ = V, Cr, Mn, Fe, Co, Ni, and Cu). It is important to emphasize that we do not conduct specific screening, but rather pay attention to the descriptors of the OER onset potential by a proper amount of calculations. An adequate linear fitting requires data points with large variability, so we use M^b^ as the active site here since in M^a^2M^b^1 systems, the ESP-derived charge (ESPC) of M^b^ is expected to be more variable than that of M^a^ relative to the monometallic counterpart (due to the relatively low contents of M^b^, more like the dopant).

**Fig. 2:**
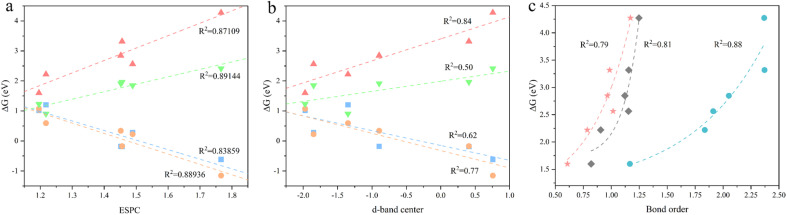
Correlation between the Gibbs energy difference and (a) ESPC, (b) d-band center and (c) bond order. Regression fittings are provided. For (a) and (b), the color blocks 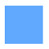, 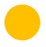, 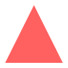 ,and 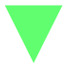 represent Δ*G*_1_, Δ*G*_2_, Δ*G*_3,_ and Δ*G*_4_, respectively. For (c), the color blocks 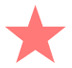, 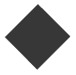, and 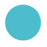 represent the M–OH bond, M–O bond, and M–OOH bond, respectively.

To obtain an accurate OER descriptive model, we comprehensively considered three most promising descriptors. They are electrostatic potential-derived charge (ESPC), density of states (DOS), and bond order (BO), which represents the spatial distribution of electrons, the energy distribution of electrons, and the chemical bond strength, respectively. Among them, we calculated the d-band center from the DOS for the convenience of quantification. For simplicity, we consider only one descriptor to correlate OER onset potentials. Additionally, we focus on the accuracy of the descriptors, while taking into account transferability, instructiveness, and computational simplicity. By correlating the Gibbs energy difference of the four primitive steps (target quantities) with the above candidates, we find that both ESPC and d-band center exhibit a linear scaling relationship with the target quantities with different accuracy (Fig. 2a and b). However, the BO shows an exponential scaling relationship with the target quantities, causing its predictive capacity drop sharply at larger values (Fig. 2c and S1†). Furthermore, the BO describing the binding strength of the adsorbed molecule to the active site cannot indicate the intrinsic properties of the material, leading to its poor transferability and instructiveness. Therefore, the BO was first excluded. For the d-band center, it requires expensive DOS calculation and may not be generalized well for MOFs possessing both metallic and semiconducting nature due to the common underestimation of the bandgap,^48^ and thus, it is not satisfactory in terms of transferability and computational simplicity. In contrast, ESPC can be obtained by fitting the charge distribution near the atom, which is computationally feasible. In addition, it is a physical quantity describing the properties of the atom with desirable transferability and instructiveness. Therefore, ESPC was finally chosen as the OER performance descriptor, and the ESPC-based OER descriptive model derived from six monometallic trigonal prismatic SBUs (Fig. 2a) is as follows:Δ*G*_1_ = −3.19ESPC + 4.81Δ*G*_2_ = −3.44ESPC + 5.07Δ*G*_3_ = 4.14ESPC − 3.12Δ*G*_4_ = 2.48ESPC − 1.84where Δ*G*_*i*_ (*i* = 1, 2, 3, 4) is the Gibbs energy difference of the elemental step in unit eV, and ESPC is the value of electrostatic potential-derived charge in unit *e*.

### 3.2 Model testing and optimization

**Fig. 3:**
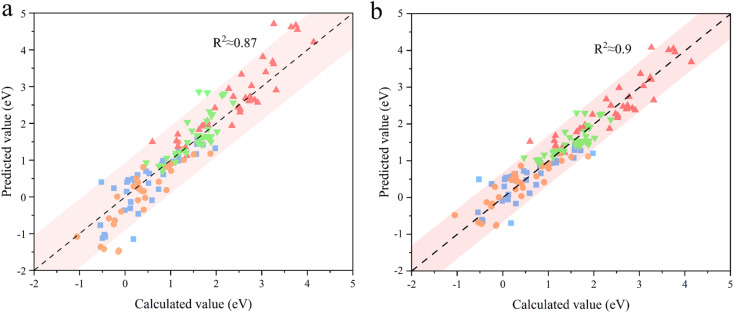
The predictive ability of the proposed model (a) before and (b) after being fed with the DFT data of SBUs with bimetallic composition. The color blocks 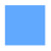, 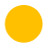, 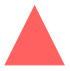 ,and 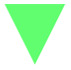 represent Δ*G*_1_, Δ*G*_2_, Δ*G*_3,_ and Δ*G*_4_, respectively. The shaded red region represents a 95% prediction interval for the linear model.

**Fig. 4:**
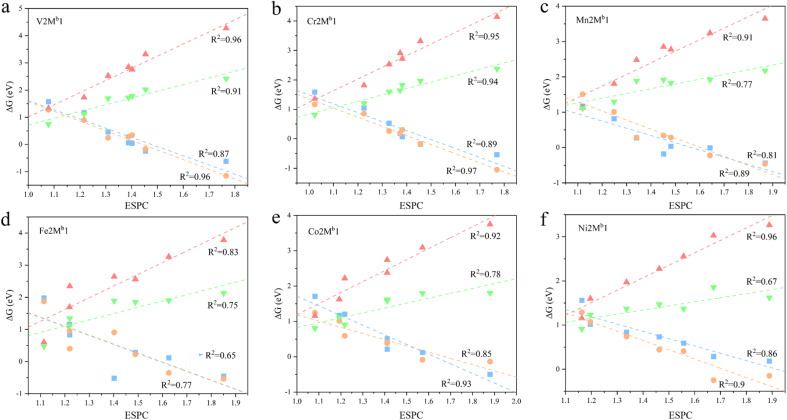
The linear scaling relationship between the Δ*G*_*i*_ (*i* = 1, 2, 3, 4) and the ESPC of the active site. (a) V2M^b^1, (b) Cr2M^b^1, (c) Mn2M^b^1, (d) Fe2M^b^1, (e) Co2M^b^1, and (f) Ni2M^b^1. The M^b^ = V, Cr, Mn, Fe, Co, Ni, and Cu. Regression fittings are provided. The four colored symbols denote 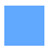: Δ*G*_1_, 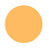: Δ*G*_2_, 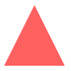: Δ*G*_3_, 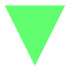: Δ*G*_4_, respectively.

The OER performance of the SBUs with bimetallic composition was predicted by the proposed model and the results were validated by DFT calculations. The minor difference between the predicted values and the validated ones indicates the great accuracy (*R*^2^ ≈ 0.87) of the descriptive model (Fig. 3a). Furthermore, the improved model shows similar predictive ability (*R*^2^ ≈ 0.90) after being fed with the DFT data of SBUs with bimetallic composition (Fig. 3b), proving the effectiveness of fitting scaling relations using monometallic SBUs. In addition, the ESPC provides an excellent description (average *R*^2^ > 0.90) of the OER performance in the case of changing the active atoms while keeping the spectator atoms consistent (Fig. 4), which is of great significance for studying the doping and active site implanting systems. Moreover, the ESPC could also rationalise the experimentally tested OER activity well (Fig. S3 and S4†). The correlations between them are similar to the predictive model raised here, indicating the potential for practical applications.

**Fig. 5:**
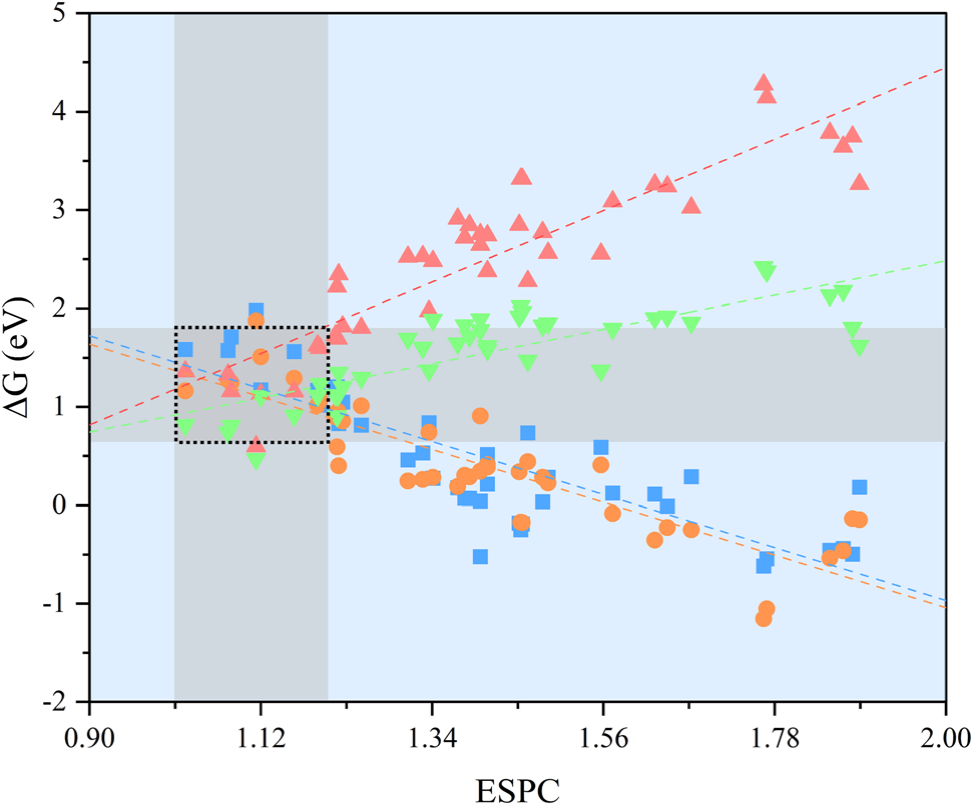
The strong linear scaling relationships between Δ*G*_*i*_ (*i* = 1, 2, 3, and 4) and the ESPC. The dashed lines provide guidance for eyes, and the shaded region represents the appropriate numerical interval of Δ*G*_*i*_ (*i* = 1, 2, 3, and 4) and the corresponding ESPC. The four colored symbols denote 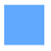: Δ*G*_1_, 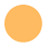: Δ*G*_2_, 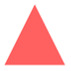: Δ*G*_3_, 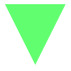: Δ*G*_4_, respectively.

Based on the proposed model, the strong linear scaling relationships between Δ*G*_*i*_ (*i* = 1, 2, 3, 4) and the ESPC are shown in Fig. 5 and Table S1,† where Δ*G*_1_ and Δ*G*_2_ are negatively correlated with the ESPC, while Δ*G*_3_ and Δ*G*_4_ show the opposite trends. This means that there is an onset potential minimum near the intersection of the four prediction lines. When the ESPC is less than 1.1, the height of the nearly coincident Δ*G*_1_ and Δ*G*_2_ prediction lines is higher than that of Δ*G*_3_ and Δ*G*_4_, indicating that the potential limiting step (PLS) is step 1 or 2 (* + H_2_O → HO* + H^+^ + e^−^ or HO* + H_2_O → O* + H^+^ + e^−^), and when the ESPC is larger than 1.1, the height of the prediction line of Δ*G*_3_ is the highest, implying that the potential limiting step is step 3 (O* + H_2_O → HOO* + H^+^ + e^−^).

**Fig. 6:**
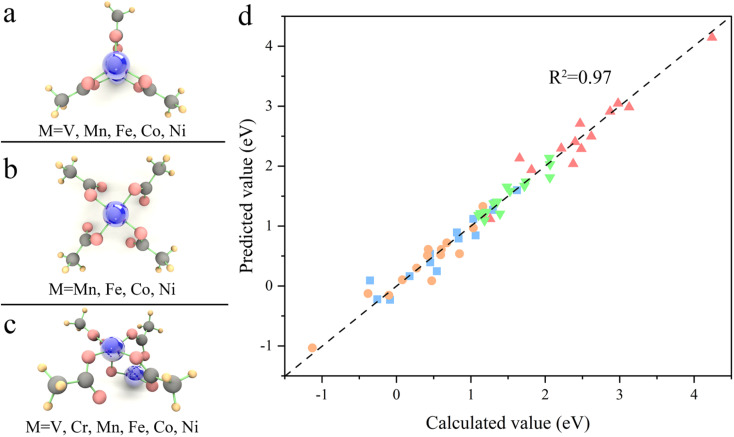
(a–c) Structures and compositions of typical SBUs. (d) Comparison between Δ*G*_*i*_ (*i* = 1, 2, 3, 4) predicted using the proposed model and Δ*G*_*i*_ (*i* = 1, 2, 3, 4) calculated using DFT for typical SBUs. The four colored symbols denote 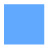: Δ*G*_1_, 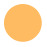: Δ*G*_2_, 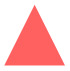: Δ*G*_3_, 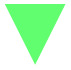: Δ*G*_4_, respectively.

Transferability is another important criterion for evaluating a descriptive model, especially for MOFs with diverse compositions and structures, as designing material often involves adjusting composition and structure. Therefore, the ESPC was further employed to predict the OER performance of the SBUs with typical 3-, 4-, and 5-coordinated configurations and representative metals (Fig. 6a–c). Excitingly, the ESPC presents a near-perfect prediction (*R*^2^ = 0.97) of the OER performance of the catalysts (Fig. 6d and Table S2†), demonstrating the general applicability of the ESPC-based descriptive model. It is pointed out that the prediction accuracy of ESPC for OER activity of typical SBUs appears to be higher than that of trigonal prismatic SBUs. This is because we introduced component orthogonality in trigonal prismatic SBUs to accurately capture the effect of bimetallic coupling on the OER activity. And as can be seen in Fig. 4, in the case of fixed M^a^ metals, the prediction accuracy of the model for OER activity of the trigonal prismatic SBUs is similar to that of the typical SBUs.

**Fig. 7:**
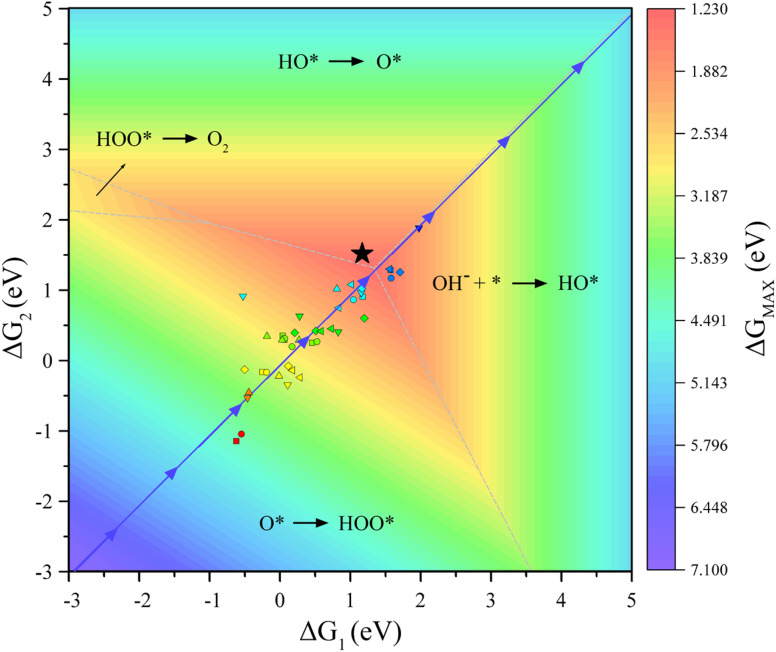
Three-dimensional volcano plot of the OER activity. The PLS on each region (divided by dashed lines) of the volcano plots is indicated. The line consisting of blue arrows represents the optimization route of OER performance guided by ESPC. The black star represents the best catalyst predicted.

In addition, our goal is to find an optimal solution over a vast material space. To validate the proposed model, a 3D volcano plot of OER activity is constructed (Fig. 7, for more details see Fig. S2†), which shows the evolution trend of the OER performance under the ESPC guidance (blue arrows). It can be clearly seen that the predicted line just passes the top of the mountain, indicating that tuning the ESPC is a desirable option for designing MOFs with high OER performance.

### 3.3 Correlation analysis of ESPC and metal combination

**Fig. 8:**
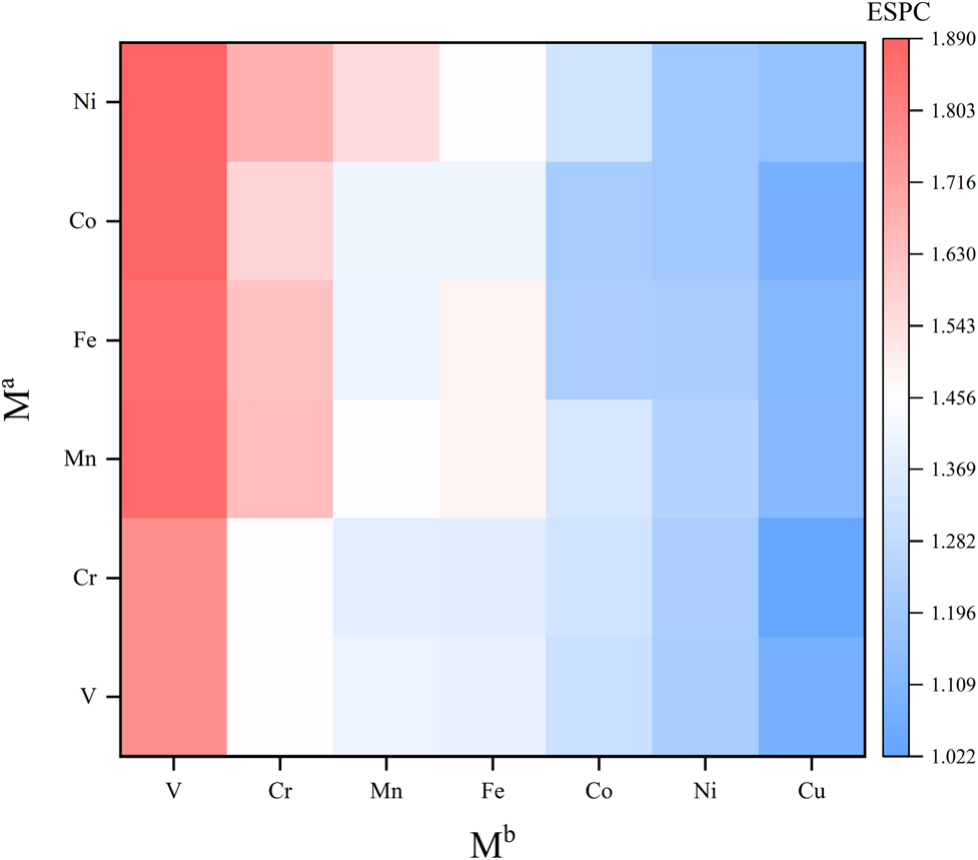
Mapping of ESPCs on the composition matrix of SBUs.

Uncovering the relationship between ESPC of the active site and metal composition is essential to fully utilize the descriptive model to guide the synthesis of high-performance materials. In view of this, we mapped the ESPC of active site onto the metal composition matrix (Fig. 8 and Table S3†). The result shows that the ESPC varies in a wide range of 1.0–1.9. When keeping the M^a^ metal unchanged, the ESPC varies with the M^b^ metals as following sequence V > Cr > Mn > Fe > Co > Ni > Cu. In addition, when M^b^ is fixed, the ESPC changes slightly with the difference of M^a^ metals, and the overall trend shifts towards larger values with the increase of the effective nuclear charge of M^a^. The matrix shows that the ESPC is in the best interval when the spectator atoms are Mn/Fe/Co/Ni, and the active site atom is Cu or Ni. Among them, Ni atoms have been widely reported as OER active sites in various catalytic materials,^49–51^ such as carbon-based materials, transition metal sulfides and MOFs. This phenomenon is consistent with the finding in this study. While very few studies report Cu as the active site for OER, thus, results in this study may shed light on the design of future Cu-based OER catalysts. More importantly, by analyzing the related experimental studies,^52–58^ we obtained the OER performance ranking for MOFs containing different elements (*i.e.*, Ni > Co > Fe, Fe > V, Fe > Mn), which is consistent with the findings of our results, demonstrating that the model proposed our work can capture the variation patterns of OER performance along with metal compositions.

**Fig. 9:**
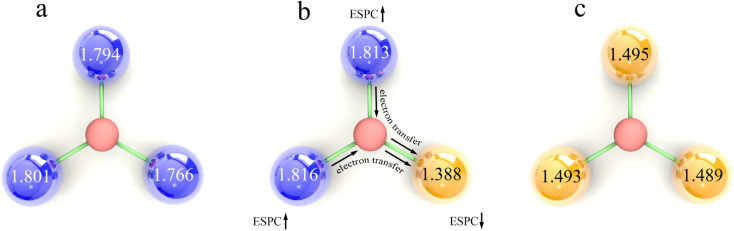
Schematic diagram of the regulation of ESPC by effective nuclear charge, taking (a) V, (b) V2Fe1, and (c) Fe as samples. HCOO fragments are omitted for clarity, in which the metals with larger effective nuclear charge, the metals with smaller effective nuclear charge and the O atoms are represented by yellow, blue and red spheres, respectively. The value of ESPCs is labeled on the models.

The variation trend of ESPC with metal composition can be explained by the difference of the element's electronegativity. For atoms in the same row of the periodic table, the distance between the valence electrons and the nucleus is similar. A bigger effective nuclear charge leads to stronger attraction to valence electrons, and the atom is less likely to lose electrons.^59,60^ In the single-metal systems, metals with larger electronegativity have higher electron retention and smaller ESPC (Fig. 9a and c). In bimetallic composition systems, the valence electrons of three metal atoms are redistributed through the μ_3_-O, and the electrons will flow to the atoms with larger electronegativity. For example, for atoms with less effective nuclear charge (like V), when they are combined with atoms with more effective nuclear charge (like Fe), their valence electrons tend to migrate to the counterpart, making the ESPC of Fe atoms in V2Fe1 less than that of Fe in the SBU with single Fe composition. In contrast, the ESPC of V atoms in V2Fe1 is larger than that of the V in the SBU with single V composition (Fig. 9b). It should be noted that this phenomenon is not distinct for systems in which Co, Ni, and Cu as M^b^, probably due to their “harder” electron cloud stemming from relatively larger electronegativity.

### 3.4 Interpretability of ESPC as an OER activity descriptor

**Fig. 10:**
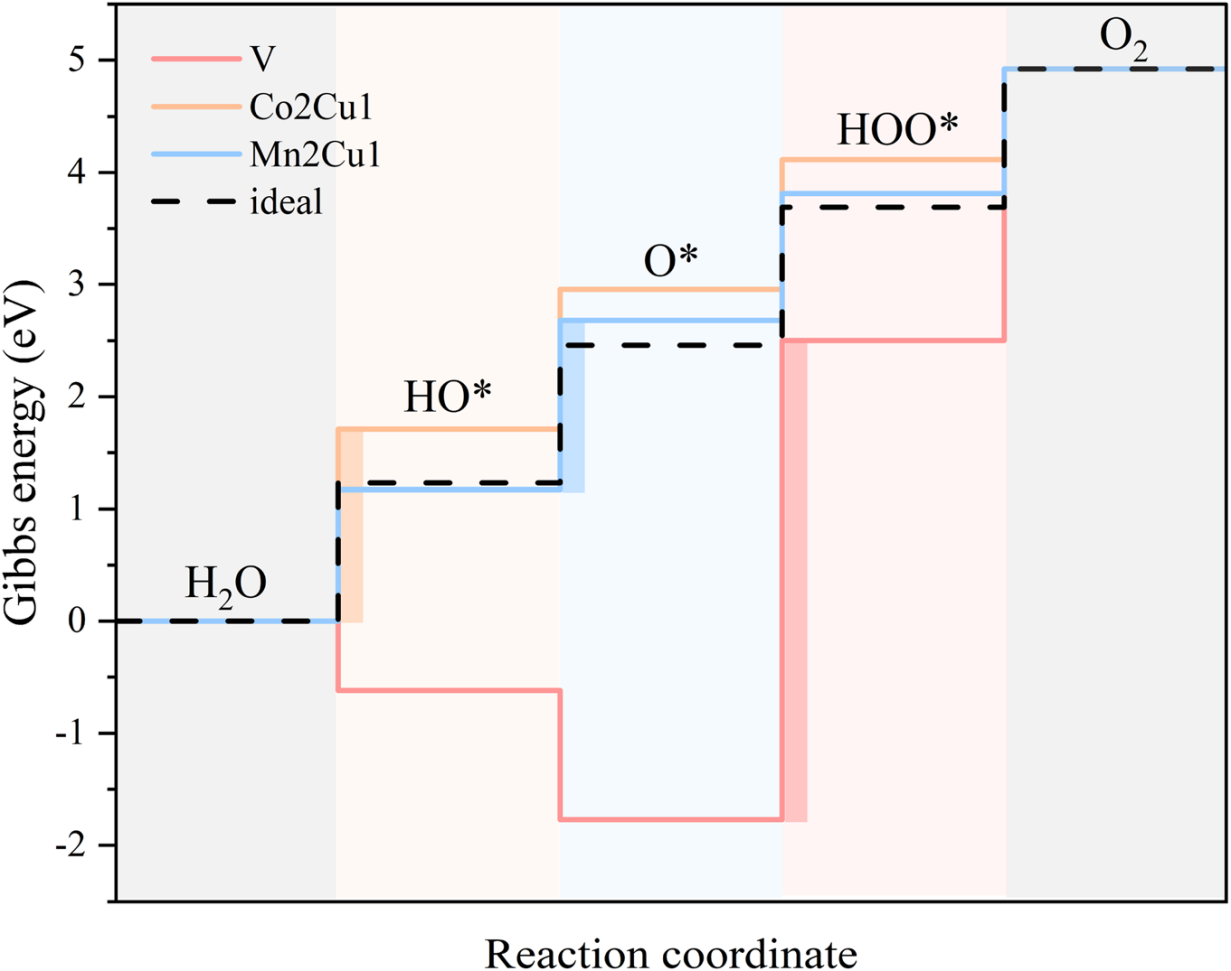
Gibbs energy diagram for V, Co2Cu1 and Mn2Cu1 systems calculated at 0 V *vs.* RHE. The color-filled rectangles denote the PLS.

Since the Δ*G*_total_ of the OER is a constant (4.92 eV), the Δ*G*_*i*_ (*i* = 1, 2, 3, 4) actually has a trade-off relationship. Taking V, Co2Cu1 and Mn2Cu1 as examples (Fig. 10), high bonding strength of V atoms with OH and O species induces excessive bonding energy, even making Δ*G*_1_ and Δ*G*_2_ become negative values. This strong adsorption leads to little activity of O, so the thermodynamic barrier between the intermediate states of V–O and V–OOH is very high, leading to an ultra-high overpotential (4.27 eV). In contrast, for Co2Cu1, the lower bonding strength of Cu atoms with OH and O facilitates the bonding of OH on Cu–O, which makes Δ*G*_1_ and Δ*G*_2_ larger and is not desirable for the entire OER process. However, for Mn2Cu1, the adsorption strength of Cu with three oxygen species is moderate, and the balanced thermodynamic barrier is beneficial for obtaining excellent OER performance.

**Fig. 11:**
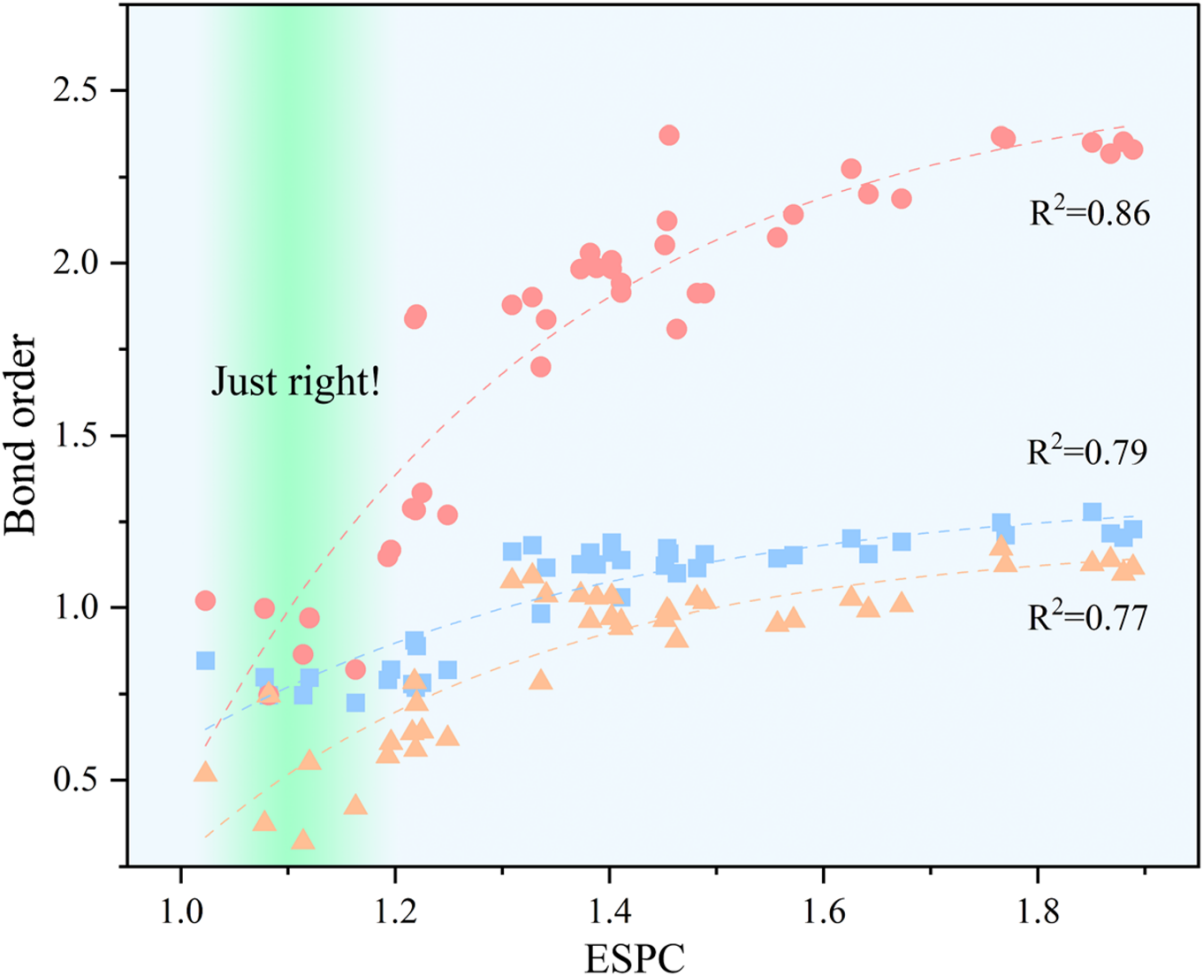
Correlation between bond order and ESPC, the dashed lines provide guidance for eyes, the color blocks 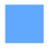, 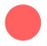, and 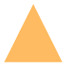 represent the M–OH bond, M–O bond, and M–OOH bond, respectively.

The relationship between ESPC and the adsorption strength of oxygen-containing intermediates can be revealed by BO. As shown in Fig. 11, with the increment of ESPC, the variation of BO_M–OH_ and BO_M–OOH_ is smaller than that of BO_M_=_O_. This could be due to the fact that M–OH and M–OOH are always single bonds, whereas the M=O bond can be either a double or a single bond with a radical character, *i.e.*, M–oxyl species. In fact, Fig. 11 shows that the bond order in M=O can adopt values ranging between *ca.* 1 and 2. The influence of the radical character in the M–O bond in OER catalysis has also been recently studied by García-Melchor *et al.*, who also proposed different scaling relations for molecular systems exhibiting M–oxo or M–oxyl characters.^61^ In addition, the BOs between active site and three oxygen-containing intermediates exist saturation values, reaching the theoretical limitation (1 for M–OH and M–OOH, and 2 for M=O) when ESPC is beyond 1.5. When ESPC is about 1.1, the BO difference among the metal–oxygen species is constant (about 0.25), which reflects the equilibrium adsorption of intermediates. When ESPC is less than 1.1, the BO_M_=_O_ decreases rapidly, which indicates that the adsorption strength of the O intermediate is weak, and the PLS prefers to occur at the first or second elemental step. When ESPC is more than 1.1, the adsorption of the O intermediate is strong, and thus, the PLS occurs at the 3rd or 4th elemental step.

**Fig. 12:**
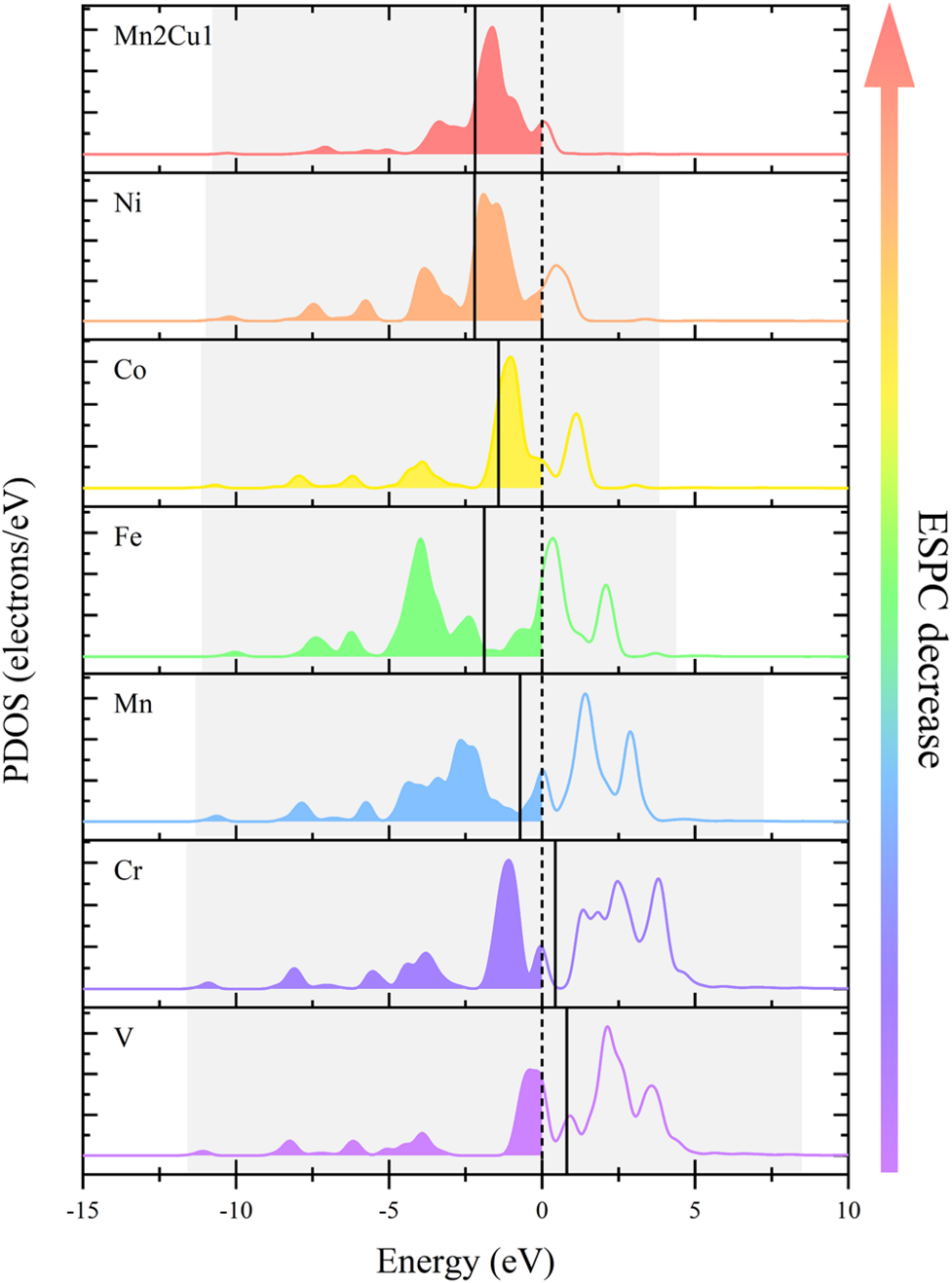
The projected density of states (PDOS) for the d-band of the active site in trigonal prismatic SBUs. The vertical dashed lines correspond to the Fermi level, which is shifted to zero and the solid lines denote the d-band centers. The occupied orbitals are filled with color.

**Fig. 13:**
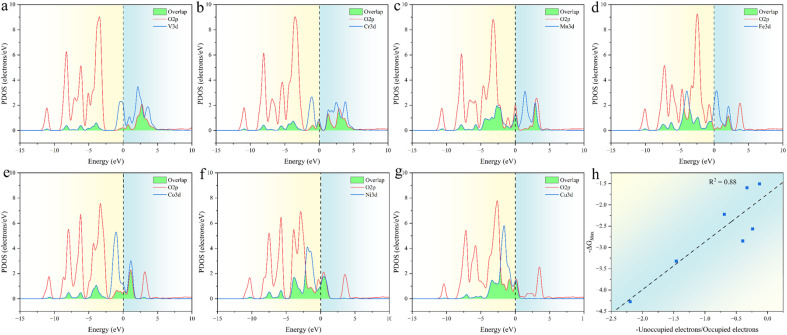
(a–g) The projected density of states (PDOS) for the 3d-orbital of the active site and the 2p-orbital of related coordinated oxygen atoms in trigonal prismatic SBUs, the vertical dashed lines correspond to the Fermi level, which is shifted to zero and the overlapping orbitals are filled with green. (h) Correlation between −Δ*G*_Max_ and −unoccupied electrons/occupied electrons, the dashed lines provide guidance for eyes.

The relationship between ESPC and the adsorption strength of oxygen-containing intermediates can be understood more deeply from the perspective of band structure. As the decrease of ESPC, the d-band center becomes progressively lower relative to the Fermi level (Fig. 12), reducing the energy of the anti-bonding state formed between the adsorbed species and the catalyst. As a result, electrons flow more easily into the antibonding state, leading to an increase in the total energy and weakening the binding strength of intermediates on the catalyst. This weakening of the binding strength is particularly pronounced for O intermediates since the double bond nature of M=O is accompanied by more electron transfer during the adsorption. In addition, with the decrease of ESPC (the active site is from V to Cu, in the order of increasing atomic number), the overall energy of the orbitals that overlapping between the metal 3d orbital and the oxygen 2p orbital (green area in Fig. 13a–g) decreases, which improves the electron filling degree of the overlapping orbitals (Fig. 13h). The stability of the metals is gradually enhanced, indicating that the “bonding ability” of the metals is weakened and leads to the reduced adsorption strength of the OER intermediate on the metals. Thus, the ESPC can be used as a simple indicator to quantify the information of the band structure, which is directly related to the OER performance.

## 4. Conclusions

In summary, we unravel that ESPC, a universal descriptor, can describe the OER activity of MOFs containing diverse SBUs very well. By analyzing the variation of ESPC with combinations of different metals, a general design principle was further put forward to enhance the OER activity of MOFs, that is, the active sites should be Ni/Cu while the spectator atoms are Mn/Fe/Co/Ni. This principle is physically meaningful since ESPC indeed is a reflection of the bonding strength between metal and oxygen-containing intermediates. The appropriate metal configuration can optimize the d-electron distribution of the active site, which reduces the height of the anti-bonding state of the metal–oxygen species and weakens the bonding strength between them as well. All these effects synergistically contribute to the high-efficiency OER process. Our work demonstrates that the regulation of metal components of SBUs is a promising strategy for developing MOF-based energy conversion devices.

## Data availability

The structures and calculated data supporting the findings of this study are available in the ESI.†

## Author contributions

Xiangdong Xue: conceptualization, methodology, software, formal analysis, investigation, data curation, writing-original draft, and visualization. Hongyi Gao: methodology, validation, writing-review & editing, supervision, project administration, and funding acquisition. Jiangtao Liu: validation and formal analysis. Ming Yang: validation and formal analysis. Shihao Feng: validation and formal analysis. Zhimeng Liu: validation and formal analysis. Jing Lin: validation and formal analysis. Jitti Kasemchainan: validation and formal analysis. Linmeng Wang: validation and formal analysis. Qilu Jia: validation and formal analysis. Ge Wang: conceptualization, resources, writing-review & editing, supervision, project administration, and funding acquisition.

## Conflicts of interest

There are no conflicts to declare.

## Supplementary Material

SC-013-D2SC04898A-s001

SC-013-D2SC04898A-s002

SC-013-D2SC04898A-s003

SC-013-D2SC04898A-s004

SC-013-D2SC04898A-s005

SC-013-D2SC04898A-s006

SC-013-D2SC04898A-s007

SC-013-D2SC04898A-s008

SC-013-D2SC04898A-s009

SC-013-D2SC04898A-s010

SC-013-D2SC04898A-s011

SC-013-D2SC04898A-s012

SC-013-D2SC04898A-s013

SC-013-D2SC04898A-s014

SC-013-D2SC04898A-s015

SC-013-D2SC04898A-s016

SC-013-D2SC04898A-s017

SC-013-D2SC04898A-s018

SC-013-D2SC04898A-s019

SC-013-D2SC04898A-s020

SC-013-D2SC04898A-s021

SC-013-D2SC04898A-s022

SC-013-D2SC04898A-s023

SC-013-D2SC04898A-s024

SC-013-D2SC04898A-s025

SC-013-D2SC04898A-s026

SC-013-D2SC04898A-s027

SC-013-D2SC04898A-s028

SC-013-D2SC04898A-s029

SC-013-D2SC04898A-s030

SC-013-D2SC04898A-s031

SC-013-D2SC04898A-s032

SC-013-D2SC04898A-s033

SC-013-D2SC04898A-s034

SC-013-D2SC04898A-s035

SC-013-D2SC04898A-s036

SC-013-D2SC04898A-s037

SC-013-D2SC04898A-s038

SC-013-D2SC04898A-s039

SC-013-D2SC04898A-s040

SC-013-D2SC04898A-s041

SC-013-D2SC04898A-s042

SC-013-D2SC04898A-s043

SC-013-D2SC04898A-s044

SC-013-D2SC04898A-s045

SC-013-D2SC04898A-s046

SC-013-D2SC04898A-s047

SC-013-D2SC04898A-s048

SC-013-D2SC04898A-s049

SC-013-D2SC04898A-s050

SC-013-D2SC04898A-s051

SC-013-D2SC04898A-s052

SC-013-D2SC04898A-s053

SC-013-D2SC04898A-s054

SC-013-D2SC04898A-s055

SC-013-D2SC04898A-s056

SC-013-D2SC04898A-s057

SC-013-D2SC04898A-s058

SC-013-D2SC04898A-s059

SC-013-D2SC04898A-s060

SC-013-D2SC04898A-s061

SC-013-D2SC04898A-s062

SC-013-D2SC04898A-s063

SC-013-D2SC04898A-s064

SC-013-D2SC04898A-s065

SC-013-D2SC04898A-s066

SC-013-D2SC04898A-s067

SC-013-D2SC04898A-s068

SC-013-D2SC04898A-s069

SC-013-D2SC04898A-s070

SC-013-D2SC04898A-s071

SC-013-D2SC04898A-s072

SC-013-D2SC04898A-s073

SC-013-D2SC04898A-s074

SC-013-D2SC04898A-s075

SC-013-D2SC04898A-s076

SC-013-D2SC04898A-s077

SC-013-D2SC04898A-s078

SC-013-D2SC04898A-s079

SC-013-D2SC04898A-s080

SC-013-D2SC04898A-s081

SC-013-D2SC04898A-s082

SC-013-D2SC04898A-s083

SC-013-D2SC04898A-s084

SC-013-D2SC04898A-s085

SC-013-D2SC04898A-s086

SC-013-D2SC04898A-s087

SC-013-D2SC04898A-s088

SC-013-D2SC04898A-s089

SC-013-D2SC04898A-s090

SC-013-D2SC04898A-s091

SC-013-D2SC04898A-s092

SC-013-D2SC04898A-s093

SC-013-D2SC04898A-s094

SC-013-D2SC04898A-s095

SC-013-D2SC04898A-s096

SC-013-D2SC04898A-s097

SC-013-D2SC04898A-s098

SC-013-D2SC04898A-s099

SC-013-D2SC04898A-s100

SC-013-D2SC04898A-s101

SC-013-D2SC04898A-s102

SC-013-D2SC04898A-s103

SC-013-D2SC04898A-s104

SC-013-D2SC04898A-s105

SC-013-D2SC04898A-s106

SC-013-D2SC04898A-s107

SC-013-D2SC04898A-s108

SC-013-D2SC04898A-s109

SC-013-D2SC04898A-s110

SC-013-D2SC04898A-s111

SC-013-D2SC04898A-s112

SC-013-D2SC04898A-s113

SC-013-D2SC04898A-s114

SC-013-D2SC04898A-s115

SC-013-D2SC04898A-s116

SC-013-D2SC04898A-s117

SC-013-D2SC04898A-s118

SC-013-D2SC04898A-s119

SC-013-D2SC04898A-s120

SC-013-D2SC04898A-s121

SC-013-D2SC04898A-s122

SC-013-D2SC04898A-s123

SC-013-D2SC04898A-s124

SC-013-D2SC04898A-s125

SC-013-D2SC04898A-s126

SC-013-D2SC04898A-s127

SC-013-D2SC04898A-s128

SC-013-D2SC04898A-s129

SC-013-D2SC04898A-s130

SC-013-D2SC04898A-s131

SC-013-D2SC04898A-s132

SC-013-D2SC04898A-s133

SC-013-D2SC04898A-s134

SC-013-D2SC04898A-s135

SC-013-D2SC04898A-s136

SC-013-D2SC04898A-s137

SC-013-D2SC04898A-s138

SC-013-D2SC04898A-s139

SC-013-D2SC04898A-s140

SC-013-D2SC04898A-s141

SC-013-D2SC04898A-s142

SC-013-D2SC04898A-s143

SC-013-D2SC04898A-s144

SC-013-D2SC04898A-s145

SC-013-D2SC04898A-s146

SC-013-D2SC04898A-s147

SC-013-D2SC04898A-s148

SC-013-D2SC04898A-s149

SC-013-D2SC04898A-s150

SC-013-D2SC04898A-s151

SC-013-D2SC04898A-s152

SC-013-D2SC04898A-s153

SC-013-D2SC04898A-s154

SC-013-D2SC04898A-s155

SC-013-D2SC04898A-s156

SC-013-D2SC04898A-s157

SC-013-D2SC04898A-s158

SC-013-D2SC04898A-s159

SC-013-D2SC04898A-s160

SC-013-D2SC04898A-s161

SC-013-D2SC04898A-s162

SC-013-D2SC04898A-s163

SC-013-D2SC04898A-s164

SC-013-D2SC04898A-s165

SC-013-D2SC04898A-s166

SC-013-D2SC04898A-s167

SC-013-D2SC04898A-s168

SC-013-D2SC04898A-s169
