# Supplementary material for: Electrostatic potential-derived charge: a universal OER performance descriptor for MOFs
Source: Chem Sci. 2022 Oct 18;13(44):13160–71. doi: 10.1039/d2sc04898a (PMC9667949; doi:10.1039/d2sc04898a)
Supplement: SC-013-D2SC04898A-s169 [file SC-013-D2SC04898A-s169.pdf]

Electronic Supplementary Material (ESI) for Chemical Science.
This journal is © The Royal Society of Chemistry 2022

Supporting Information

Electrostatic potential-derived charge: a universal OER performance descriptor for MOFs

Xiangdong Xue[a], Hongyi Gao[a,*], Jiangtao Liu[c], Ming Yang[d], Shihao Feng[a], Zhimeng Liu[a], Jing Lin[a], Jitti Kasemchainan[e], Linmeng Wang[a], Qilu Jia[a], Ge Wang[a,b,*]

[a] *Beijing Advanced Innovation Center for Materials Genome Engineering, Beijing Key Laboratory of Function Materials for Molecule & Structure Construction, School of Materials Science and Engineering, University of Science and Technology Beijing, Beijing, 100083, PR China*

[b] *Shunde Graduate School, University of Science and Technology Beijing, Shunde, 528399, PR China*

[c] *State Key Laboratory of Advanced Chemical Power Sources. Guizhou Meiling Power Sources Co., Ltd., Zunyi, Guizhou 563003, PR China*

[d] *Department of Applied Physics, The Hong Kong Polytechnic University, Hung Hom, Hong Kong SAR, China*

[e] *Department of Chemical Technology, Chulalongkorn University, Bangkok 10330, Thailand*

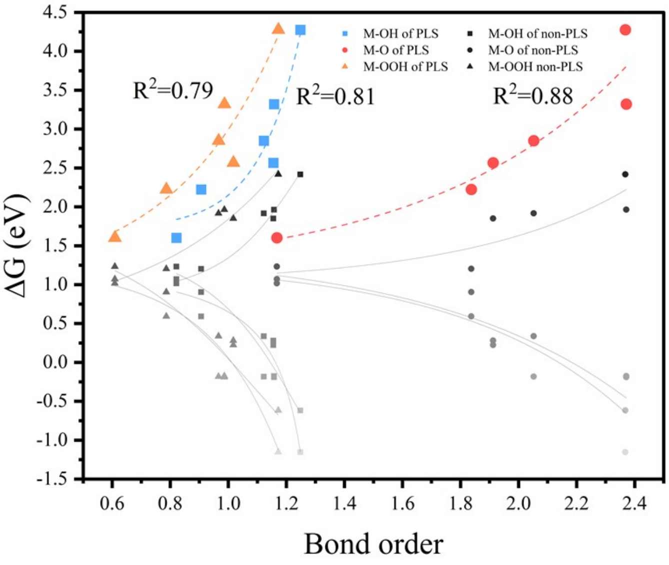

Figure S1. Correlation between the Gibbs energy difference and bond order. All data are fitted to exponential functions.

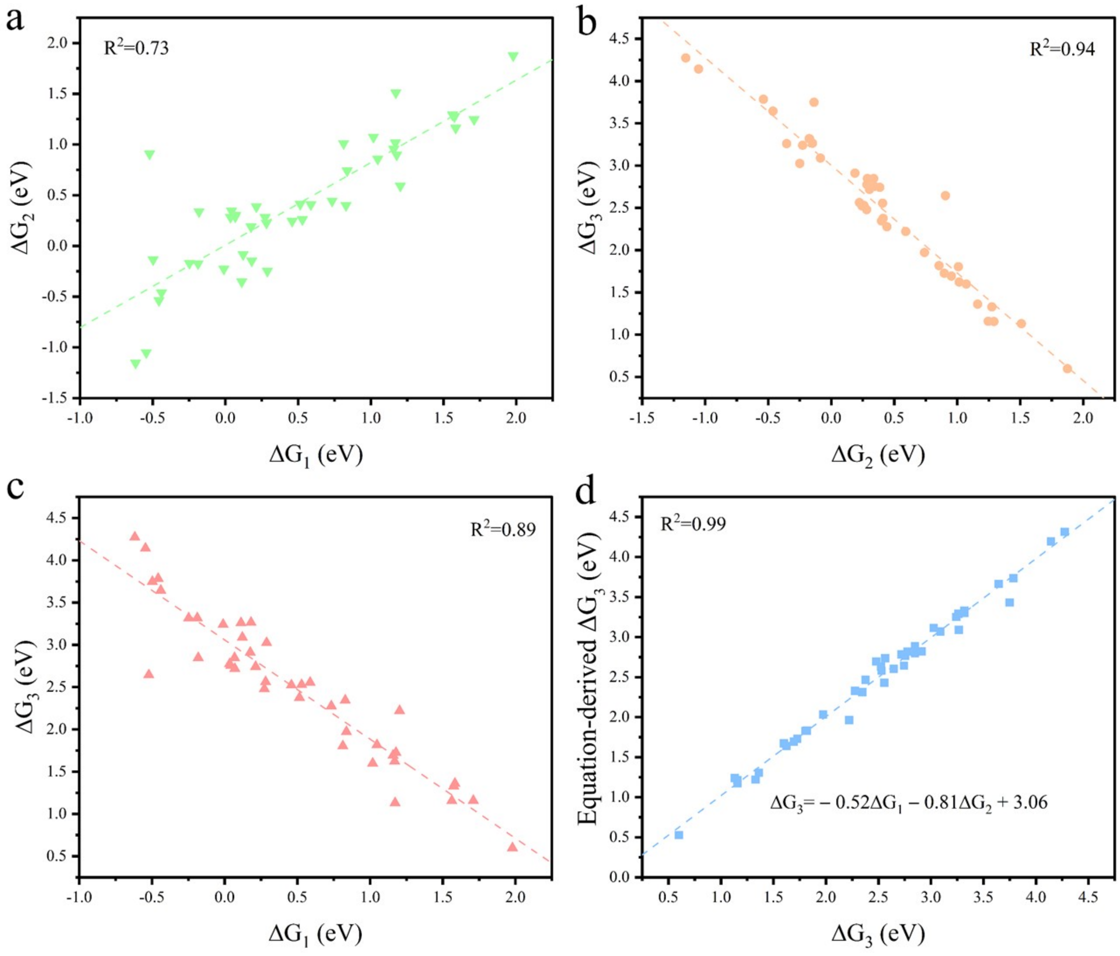

Figure S2. The relationship between the ΔG_1, ΔG_2 and ΔG_3 in the OER process. Dash lines were fitted for all data represented.

A critical indicator for evaluating the activity of an electrocatalyst is the reaction potential, which can be represented by the Gibbs energy difference (ΔG) reflecting the energy barrier of a chemical reaction. For four-electron OER process, the ΔG_i (i=1, 2, 3, 4) reflect the energy barrier of each elemental step. Nevertheless, it is complicating to use ΔG_i (i=1, 2, 3, 4) to evaluate the OER performance of the catalyst. It is worthy to clarify the relationship between the ΔG_i (i=1, 2, 3, 4) to simplify the complexity of performance ranking. Firstly, we analyzed the scaling relationship between ΔG_1, ΔG_2 and ΔG_3. ΔG_4 is not considered because it is not an independent quantity under the condition that the total Gibbs energy difference (ΔG_{total}) of OER is 4.92 eV[1]. The obvious linear relationships between ΔG_1, ΔG_2 and ΔG_3 were shown in Figure S2a-c, where ΔG_1 and ΔG_2 are positively correlated, and they are all negatively correlated with ΔG_3. Moreover, although ΔG_3 can be approximately replaced by a constant subtract ΔG_2 ($R^2 = 0.94$) as reported in other literature[2], by multiple linear regression analysis, ΔG_3 can almost be represented by a formula containing ΔG_1 and ΔG_2 ($R^2 = 0.99$, Figure S2d). And because of the non-independence of ΔG_4, the ΔG_i (i=1, 2, 3, 4) can be quantified by ΔG_1 and ΔG_2.

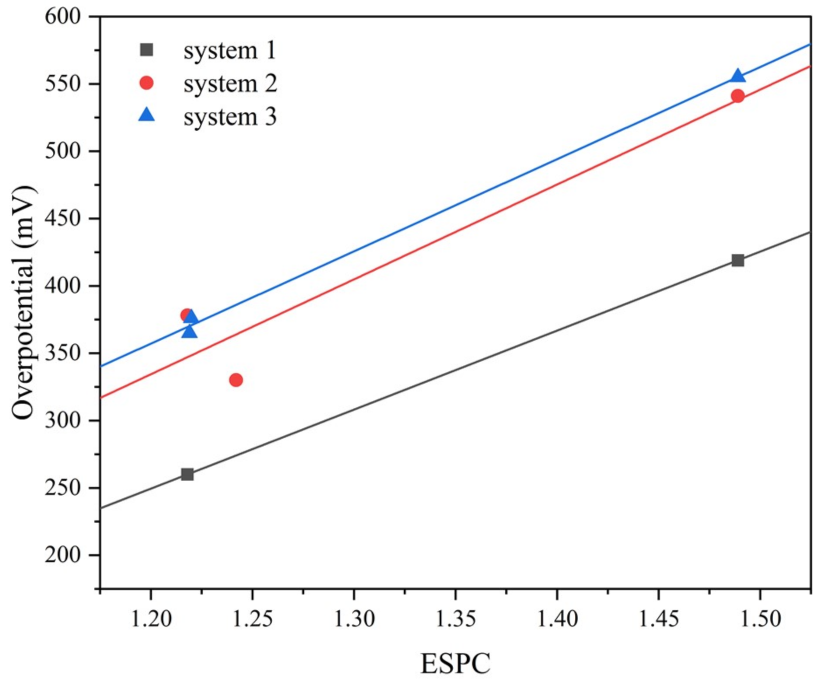

Figure S3 The correlations between experiment-derived overpotential and ESPC, system 1 comes from ref. 3, system 2 comes from ref. 4, and system 3 comes from ref. 5.

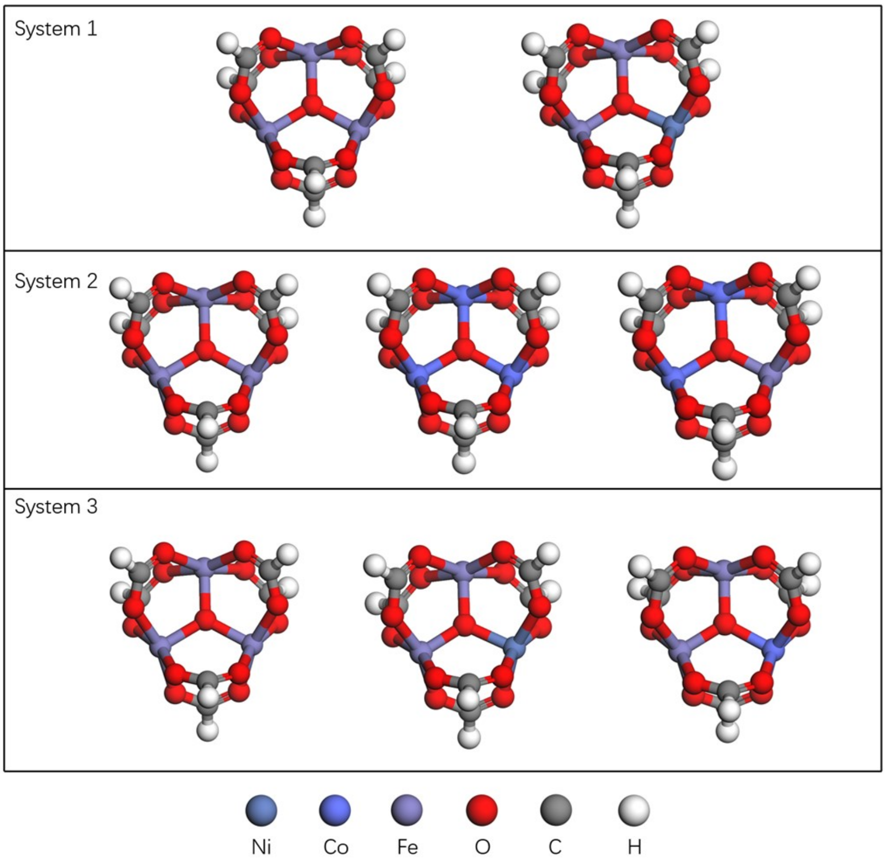

Figure S4 The structure using for calculation in figure S3.

Table S1. The original calculated OER data of MOFs containing triagonal prismatic SBUs

Configuration		E_{DFT} (eV)	E_{ZPE} (eV)	TS (eV)	ΔG (eV)
V	Cat.	-39939.90			
	HO-Cat.	-42002.46	0.34	0.10	-0.62
	O-Cat.	-41987.45	0.08	0.06	-1.15
	HOO-Cat.	-44045.12	0.44	0.19	4.27
	O_2+Cat.				2.42
Cr	Cat.	-41334.73			
	HO-Cat.	-43396.86	0.34	0.11	-0.19
	O-Cat.	-43380.89	0.08	0.05	-0.17
	HOO-Cat.	-45439.48	0.43	0.20	3.32
	O_2+Cat.				1.96
Mn	Cat.	-42846.95			
	HO-Cat.	-44909.08	0.34	0.10	-0.18
	O-Cat.	-44892.59	0.08	0.05	0.34
	HOO-Cat.	-46951.68	0.45	0.18	2.85
	O_2+Cat.				1.92
Fe	Cat.	-44592.81			
	HO-Cat.	-46654.50	0.36	0.09	0.28
	O-Cat.	-46638.09	0.07	0.05	0.22
	HOO-Cat.	-48697.44	0.44	0.21	2.56
	O_2+Cat.				1.85
Co	Cat.	-46518.61			
	HO-Cat.	-48579.38	0.36	0.08	1.20
	O-Cat.	-48562.59	0.07	0.05	0.59
	HOO-Cat.	-50622.35	0.45	0.16	2.22
	O_2+Cat.				0.91
Ni	Cat.	-48692.90			
	HO-Cat.	-50753.83	0.35	0.11	1.02
	O-Cat.	-50736.58	0.06	0.06	1.07
	HOO-Cat.	-52796.95	0.44	0.17	1.60
	O_2+Cat.				1.23
V2Cu1_Cu	Cat.	-43646.29			
	HO-Cat.	-45706.65	0.34	0.11	1.58
	O-Cat.	-45689.16	0.04	0.09	1.28
	HOO-Cat.	-47749.80	0.44	0.21	1.33
	O_2+Cat.				0.74
V2Mn1_Mn	Cat.	-40909.19			
	HO-Cat.	-42971.11	0.35	0.10	0.04
	O-Cat.	-42954.59	0.07	0.05	0.34
	HOO-Cat.	-45013.78	0.45	0.18	2.75
	O_2+Cat.				1.78

Table S1. (*continued*)

Configuration		E_{DFT} (eV)	E_{ZPE} (eV)	TS (eV)	ΔG(eV)
V2Cr1_Cr	Cat.	-40404.87			
	HO-Cat.	-42467.06	0.35	0.10	-0.25
	O-Cat.	-42451.07	0.08	0.06	-0.17
	HOO-Cat.	-44509.68	0.44	0.19	3.32
	O_2+Cat.				2.02
V2Fe1_Fe	Cat.	-41491.10			
	HO-Cat.	-43552.97	0.34	0.10	0.07
	O-Cat.	-43536.54	0.08	0.05	0.29
	HOO-Cat.	-45595.63	0.44	0.17	2.85
	O_2+Cat.				1.72
V2Co1_Co	Cat.	-42132.72			
	HO-Cat.	-44194.25	0.37	0.08	0.46
	O-Cat.	-44177.79	0.07	0.06	0.24
	HOO-Cat.	-46237.26	0.46	0.15	2.52
	O_2+Cat.				1.69
V2Ni1_Ni	Cat.	-42858.41			
	HO-Cat.	-44919.19	0.35	0.09	1.18
	O-Cat.	-44902.08	0.05	0.07	0.90
	HOO-Cat.	-46962.34	0.45	0.18	1.73
	O_2+Cat.				1.12
Cr2V1_V	Cat.	-40869.85			
	HO-Cat.	-42932.36	0.35	0.09	-0.54
	O-Cat.	-42917.23	0.08	0.06	-1.05
	HOO-Cat.	-44975.02	0.44	0.19	4.14
	O_2+Cat.				2.37
Cr2Mn1_Mn	Cat.	-41839.05			
	HO-Cat.	-43900.93	0.35	0.10	0.07
	O-Cat.	-43884.46	0.07	0.05	0.30
	HOO-Cat.	-45943.68	0.44	0.18	2.72
	O_2+Cat.				1.83
Cr2Fe1_Fe	Cat.	-42420.98			
	HO-Cat.	-44482.75	0.34	0.10	0.18
	O-Cat.	-44466.40	0.07	0.05	0.19
	HOO-Cat.	-46525.46	0.45	0.16	2.91
	O_2+Cat.				1.64
Cr2Co1_Co	Cat.	-43062.57			
	HO-Cat.	-45124.03	0.36	0.08	0.53
	O-Cat.	-45107.56	0.07	0.05	0.26
	HOO-Cat.	-47167.04	0.46	0.14	2.53
	O_2+Cat.				1.60

Table S1. (*continued*)

Configuration		E_{DFT} (eV)	E_{ZPE} (eV)	TS (eV)	ΔG (eV)
Cr2Ni1_Ni	Cat.	-43788.01			
	HO-Cat.	-45848.93	0.36	0.09	1.05
	O-Cat.	-45831.85	0.05	0.07	0.86
	HOO-Cat.	-47892.03	0.45	0.17	1.82
	O_2+Cat.				1.20
Cr2Cu1_Cu	Cat.	-44575.97			
	HO-Cat.	-46636.34	0.35	0.10	1.58
	O-Cat.	-46618.95	0.05	0.08	1.16
	HOO-Cat.	-48679.56	0.43	0.20	1.36
	O_2+Cat.				0.81
Mn2V1_V	Cat.	-41878.45			
	HO-Cat.	-43940.84	0.35	0.09	-0.44
	O-Cat.	-43925.13	0.08	0.06	-0.46
	HOO-Cat.	-45983.48	0.47	0.16	3.64
	O_2+Cat.				2.18
Mn2Cr1_Cr	Cat.	-42343.07			
	HO-Cat.	-44405.04	0.35	0.10	-0.01
	O-Cat.	-44389.10	0.08	0.05	-0.23
	HOO-Cat.	-46447.83	0.46	0.17	3.24
	O_2+Cat.				1.92
Fe2V1_V	Cat.	-43042.27			
	HO-Cat.	-45104.68	0.35	0.09	-0.46
	O-Cat.	-45089.05	0.08	0.06	-0.54
	HOO-Cat.	-47147.20	0.45	0.20	3.78
	O_2+Cat.				2.13
Mn2Fe1_Fe	Cat.	-43428.90			
	HO-Cat.	-45490.81	0.35	0.10	0.03
	O-Cat.	-45474.37	0.07	0.05	0.28
	HOO-Cat.	-47533.55	0.45	0.16	2.78
	O_2+Cat.				1.83
Mn2Co1_Co	Cat.	-44070.43			
	HO-Cat.	-46132.15	0.37	0.08	0.27
	O-Cat.	-46115.66	0.07	0.05	0.28
	HOO-Cat.	-48175.16	0.46	0.16	2.48
	O_2+Cat.				1.89
Mn2Ni1_Ni	Cat.	-44795.82			
	HO-Cat.	-46856.96	0.35	0.10	0.81
	O-Cat.	-46839.74	0.05	0.07	1.01
	HOO-Cat.	-48899.93	0.45	0.17	1.80
	O_2+Cat.				1.29

Table S1. (*continued*)

Configuration		E_{DFT} (eV)	E_{ZPE} (eV)	TS (eV)	ΔG (eV)
Mn2Cu1_Cu	Cat.	-45583.74			
	HO-Cat.	-47644.51	0.34	0.10	1.17
	O-Cat.	-47626.79	0.05	0.08	1.51
	HOO-Cat.	-49687.65	0.44	0.18	1.13
	O_2+Cat.				1.11
Fe2Cr1_Cr	Cat.	-43506.97			
	HO-Cat.	-45568.83	0.35	0.09	0.11
	O-Cat.	-45553.00	0.08	0.05	-0.35
	HOO-Cat.	-47611.72	0.46	0.16	3.26
	O_2+Cat.				1.90
Fe2Mn1_Mn	Cat.	-44010.94			
	HO-Cat.	-46073.42	0.35	0.09	-0.52
	O-Cat.	-46056.33	0.07	0.05	0.91
	HOO-Cat.	-48115.67	0.46	0.16	2.64
	O_2+Cat.				1.89
Fe2Co1_Co	Cat.	-45234.73			
	HO-Cat.	-47295.89	0.37	0.08	0.83
	O-Cat.	-47279.28	0.07	0.05	0.40
	HOO-Cat.	-49338.87	0.45	0.20	2.35
	O_2+Cat.				1.35
Fe2Ni1_Ni	Cat.	-45959.92			
	HO-Cat.	-48020.73	0.35	0.09	1.16
	O-Cat.	-48003.55	0.05	0.07	0.95
	HOO-Cat.	-50063.85	0.45	0.17	1.69
	O_2+Cat.				1.11
Fe2Cu1_Cu	Cat.	-46748.05			
	HO-Cat.	-48807.99	0.33	0.11	1.98
	O-Cat.	-48789.91	0.04	0.08	1.88
	HOO-Cat.	-50851.29	0.43	0.21	0.60
	O_2+Cat.				0.47
Co2V1_V	Cat.	-44325.84			
	HO-Cat.	-46388.29	0.35	0.09	-0.50
	O-Cat.	-46372.25	0.08	0.06	-0.14
	HOO-Cat.	-48430.49	0.46	0.16	3.75
	O_2+Cat.				1.80
Co2Cr1_Cr	Cat.	-44790.37			
	HO-Cat.	-46852.21	0.35	0.09	0.12
	O-Cat.	-46836.12	0.08	0.05	-0.08
	HOO-Cat.	-48894.99	0.46	0.18	3.09
	O_2+Cat.				1.79

Table S1. (*continued*)

Configuration		E_{DFT} (eV)	E_{ZPE} (eV)	TS (eV)	ΔG (eV)
Co2Mn1_Mn	Cat.	-45294.42			
	HO-Cat.	-47356.17	0.35	0.09	0.21
	O-Cat.	-47339.60	0.07	0.05	0.39
	HOO-Cat.	-49398.83	0.45	0.17	2.74
	O_2+Cat.				1.58
Co2Fe1_Fe	Cat.	-45876.54			
	HO-Cat.	-47937.97	0.34	0.10	0.51
	O-Cat.	-47921.39	0.07	0.05	0.41
	HOO-Cat.	-49981.01	0.46	0.15	2.38
	O_2+Cat.				1.62
Co2Ni1_Ni	Cat.	-47243.29			
	HO-Cat.	-49304.11	0.36	0.08	1.17
	O-Cat.	-49286.86	0.06	0.06	1.02
	HOO-Cat.	-51347.23	0.45	0.17	1.62
	O_2+Cat.				1.11
Co2Cu1_Cu	Cat.	-48030.98			
	HO-Cat.	-50091.21	0.34	0.10	1.71
	O-Cat.	-50073.71	0.05	0.12	1.25
	HOO-Cat.	-52134.56	0.44	0.20	1.16
	O_2+Cat.				0.81
Ni2V1_V	Cat.	-45776.39			
	HO-Cat.	-47838.17	0.35	0.09	0.18
	O-Cat.	-47822.14	0.08	0.06	-0.15
	HOO-Cat.	-49880.86	0.46	0.16	3.26
	O_2+Cat.				1.62
Ni2Cr1_Cr	Cat.	-46240.55			
	HO-Cat.	-48302.23	0.35	0.09	0.29
	O-Cat.	-48286.29	0.08	0.05	-0.25
	HOO-Cat.	-50345.25	0.46	0.16	3.03
	O_2+Cat.				1.86
Ni2Mn1_Mn	Cat.	-46744.90			
	HO-Cat.	-48806.29	0.36	0.08	0.59
	O-Cat.	-48789.70	0.08	0.05	0.41
	HOO-Cat.	-50849.12	0.46	0.15	2.56
	O_2+Cat.				1.37
Ni2Fe1_Fe	Cat.	-47326.67			
	HO-Cat.	-49387.90	0.36	0.09	0.73
	O-Cat.	-49371.28	0.07	0.05	0.44
	HOO-Cat.	-51430.98	0.46	0.15	2.28
	O_2+Cat.				1.47

Table S1. (*continued*)

Configuration		E_{DFT} (eV)	E_{ZPE} (eV)	TS (eV)	ΔG (eV)
Ni2Co1_Co	Cat.	-47968.15			
	HO-Cat.	-50029.30	0.36	0.08	0.84
	O-Cat.	-50012.36	0.07	0.05	0.74
	HOO-Cat.	-52072.36	0.45	0.16	1.97
	O_2+Cat.				1.37
Ni2Cu1_Cu	Cat.	-49480.49			
	HO-Cat.	-51540.84	0.33	0.12	1.56
	O-Cat.	-51523.37	0.04	0.08	1.29
	HOO-Cat.	-53584.20	0.44	0.19	1.16
	O_2+Cat.				0.91

Table S2. The original calculated OER data of MOFs containing typical SBUs.

Configuration		E_{DFT} (eV)	E_{ZPE} (eV)	TS (eV)	ΔG (eV)
4-coordinated_Mn	Cat.	-28157.21			
	HO-Cat.	-30218.33	0.35	0.09	0.84
	O-Cat.	-30202.28	0.08	0.05	-0.10
	HOO-Cat.	-32261.25	0.45	0.16	2.98
	O_2+Cat.				1.21
4-coordinated_Fe	Cat.	-28739.07			
	HO-Cat.	-30799.97	0.34	0.11	1.03
	O-Cat.	-30783.55	0.07	0.05	0.27
	HOO-Cat.	-32843.00	0.44	0.18	2.49
	O_2+Cat.				1.13
4-coordinated_Co	Cat.	-29380.20			
	HO-Cat.	-31440.91	0.37	0.07	1.30
	O-Cat.	-31424.27	0.07	0.06	0.42
	HOO-Cat.	-33484.41	0.41	0.14	1.82
	O_2+Cat.				1.39
4-coordinated_Ni	Cat.	-30105.31			
	HO-Cat.	-32165.63	0.35	0.11	1.62
	O-Cat.	-32148.39	0.05	0.08	1.03
	HOO-Cat.	-34209.17	0.39	0.17	1.16
	O_2+Cat.				1.10
3-coordinated_V	Cat.	-35835.77			
	HO-Cat.	-37899.44	0.34	0.11	-1.73
	O-Cat.	-37884.79	0.07	0.07	-1.52
	HOO-Cat.	-39942.28	0.42	0.21	4.42
	O_2+Cat.				3.75
3-coordinated_Mn	Cat.	-37776.93			
	HO-Cat.	-39838.96	0.34	0.10	-0.09
	O-Cat.	-39822.68	0.07	0.08	0.08
	HOO-Cat.	-41881.82	0.46	0.17	2.87
	O_2+Cat.				2.05
3-coordinated_Fe	Cat.	-38941.23			
	HO-Cat.	-41003.45	0.32	0.16	-0.36
	O-Cat.	-40986.72	0.06	0.09	0.59
	HOO-Cat.	-43046.05	0.43	0.22	2.62
	O_2+Cat.				2.06
3-coordinated_Co	Cat.	-40224.30			
	HO-Cat.	-42286.02	0.33	0.13	0.18
	O-Cat.	-42269.24	0.05	0.09	0.60
	HOO-Cat.	-44328.81	0.44	0.21	2.40
	O_2+Cat.				1.74

Table S2. (*continued*)

Configuration		E_{DFT} (eV)	E_{ZPE} (eV)	TS (eV)	ΔG (eV)
3-coordinated_Ni	Cat.	-41674.52			
	HO-Cat.	-43735.91	0.35	0.12	0.54
	O-Cat.	-43719.02	0.05	0.10	0.68
	HOO-Cat.	-45778.74	0.43	0.24	2.22
	O_2+Cat.				1.49
5-coordinated_V	Cat.	-31579.68			
	HO-Cat.	-33641.89	0.35	0.09	-0.26
	O-Cat.	-33626.86	0.08	0.05	-1.13
	HOO-Cat.	-35684.57	0.46	0.17	4.24
	O_2+Cat.				2.07
5-coordinated_Cr	Cat.	-32509.52			
	HO-Cat.	-34571.02	0.34	0.10	0.45
	O-Cat.	-34555.25	0.08	0.05	-0.38
	HOO-Cat.	-36614.08	0.45	0.16	3.13
	O_2+Cat.				1.72
5-coordinated_Mn	Cat.	-33517.86			
	HO-Cat.	-35579.39	0.36	0.08	0.45
	O-Cat.	-35562.73	0.08	0.05	0.47
	HOO-Cat.	-37622.22	0.45	0.16	2.47
	O_2+Cat.				1.53
5-coordinated_Fe	Cat.	-34681.79			
	HO-Cat.	-36742.97	0.36	0.08	0.81
	O-Cat.	-36726.34	0.07	0.05	0.43
	HOO-Cat.	-38785.95	0.46	0.15	2.38
	O_2+Cat.				1.30
5-coordinated_Co	Cat.	-35964.90			
	HO-Cat.	-38025.82	0.36	0.08	1.06
	O-Cat.	-38008.77	0.07	0.05	0.85
	HOO-Cat.	-40069.10	0.45	0.15	1.65
	O_2+Cat.				1.35
5-coordinated_Ni	Cat.	-37414.79			
	HO-Cat.	-39475.45	0.36	0.08	1.31
	O-Cat.	-39458.07	0.05	0.06	1.16
	HOO-Cat.	-41518.81	0.44	0.15	1.26
	O_2+Cat.				1.18

Table S3. The active-site ESPCs of SBUs with different metal combinations.

	V	Cr	Mn	Fe	Co	Ni	Cu
V	1.766	1.454	1.402	1.388	1.309	1.216	1.078
Cr	1.77	1.456	1.382	1.373	1.328	1.225	1.023
Mn	1.868	1.642	1.452	1.482	1.341	1.249	1.12
Fe	1.851	1.626	1.402	1.489	1.22	1.219	1.114
Co	1.88	1.572	1.411	1.411	1.218	1.193	1.082
Ni	1.889	1.673	1.557	1.463	1.336	1.196	1.163

REFERENCES

[1] Y. Zhou, G. Gao, W. Chu, L.-W. Wang, Transition-metal single atoms embedded into defective BC 3 as efficient electrocatalysts for oxygen evolution and reduction reactions, Nanoscale 13 (2021) 1331-1339.

[2] M.J. Craig, G. Coulter, E. Dolan, J. Soriano-López, E. Mates-Torres, W. Schmitt, M. García-Melchor, Universal scaling relations for the rational design of molecular water oxidation catalysts with near-zero overpotential, Nat. Commun. 10 (2019) 1-9.

[3] J. Li, W. Huang, M. Wang, S. Xi, J. Meng, K. Zhao, J. Jin, W. Xu, Z. Wang, X. Liu, Low-crystalline bimetallic metal-organic framework electrocatalysts with rich active sites for oxygen evolution, ACS Energy Lett. 4 (2018) 285-292.

[4] Z. Xue, Y. Li, Y. Zhang, W. Geng, B. Jia, J. Tang, S. Bao, H.P. Wang, Y. Fan, Z.w. Wei, Modulating electronic structure of metal-organic framework for efficient electrocatalytic oxygen evolution, Adv. Energy Mater. 8 (2018) 1801564.

[5] X.L. Wang, L.Z. Dong, M. Qiao, Y.J. Tang, J. Liu, Y. Li, S.L. Li, J.X. Su, Y.Q. Lan, Exploring the performance improvement of the oxygen evolution reaction in a stable bimetal-organic framework system, Angew. Chem. Int. Ed. 57 (2018) 9660-9664.
